# Identification of Human S100A9 as a Novel Target for Treatment of Autoimmune Disease via Binding to Quinoline-3-Carboxamides

**DOI:** 10.1371/journal.pbio.1000097

**Published:** 2009-04-28

**Authors:** Per Björk, Anders Björk, Thomas Vogl, Martin Stenström, David Liberg, Anders Olsson, Johannes Roth, Fredrik Ivars, Tomas Leanderson

**Affiliations:** 1 Active Biotech AB, Lund, Sweden; 2 Institute of Immunology, University of Münster, Münster, Germany; 3 Immunology Group, Lund University, Lund, Sweden; Osaka University, Japan

## Abstract

Despite more than 25 years of research, the molecular targets of quinoline-3-carboxamides have been elusive although these compounds are currently in Phase II and III development for treatment of autoimmune/inflammatory diseases in humans. Using photoaffinity cross-linking of a radioactively labelled quinoline-3-carboxamide compound, we could determine a direct association between human S100A9 and quinoline-3-carboxamides. This interaction was strictly dependent on both Zn^++^ and Ca^++^. We also show that S100A9 in the presence of Zn^++^ and Ca^++^ is an efficient ligand of receptor for advanced glycation end products (RAGE) and also an endogenous Toll ligand in that it shows a highly specific interaction with TLR4/MD2. Both these interactions are inhibited by quinoline-3-carboxamides. A clear structure-activity relationship (SAR) emerged with regard to the binding of quinoline-3-carboxamides to S100A9, as well as these compounds potency to inhibit interactions with RAGE or TLR4/MD2. The same SAR was observed when the compound's ability to inhibit acute experimental autoimmune encephalomyelitis in mice in vivo was analysed. Quinoline-3-carboxamides would also inhibit TNFα release in a S100A9-dependent model in vivo, as would antibodies raised against the quinoline-3-carboxamide–binding domain of S100A9. Thus, S100A9 appears to be a focal molecule in the control of autoimmune disease via its interactions with proinflammatory mediators. The specific binding of quinoline-3-carboxamides to S100A9 explains the immunomodulatory activity of this class of compounds and defines S100A9 as a novel target for treatment of human autoimmune diseases.

## Author Summary

What molecules and mechanisms underlie the development of autoimmune diseases such as multiple sclerosis, rheumatoid arthritis, and systemic lupus erythematosus are largely unknown. To gain some insight into the process, we use a class of chemical compounds, quinoline-3-carboxamides (Q compounds), which modify disease in both experimental animal models and in clinical trials, but whose target(s) have been elusive. We show that these Q compounds bind to a molecule called S100A9 that is expressed on the surface of various monocyte populations in the peripheral blood. Furthermore, we show that Q compounds inhibit the interaction of S100A9 with two well-known proinflammatory receptors (the Toll-like receptor 4 [TLR4] and receptor of advanced glycation end products [RAGE]). We provide a missing piece to the puzzle in that we identify S100A9 as a target of Q compound drugs and identify a new mechanism where S100A9 promotes inflammation at early stages of immune activation and thereby a role in the development of autoimmune disease.

## Abstract 3

A target molecule is identified for the quinoline-3-carboxamide drugs famed for their ability to alleviate autoimmunity. The role of S100A9 in promoting inflammation is described, as well as that of the pro-inflammatory receptors with which it interacts.

## Introduction

The medical need for novel treatments of human autoimmune/inflammatory disease is high. Quinoline-3-carboxamides (Q compounds) have been explored as treatments for autoimmune/inflammatory diseases in humans. They have shown proof-of-concept in clinical trials for the treatment of multiple sclerosis (MS) [1–4] and Type I diabetes [5], and are currently in Phase III clinical development for the treatment of MS [6] and are about to enter Phase II for the treatment of systemic lupus erythematosus (SLE). The target molecule and the mode of action of this class of compounds have remained unknown for over 25 years. Q compounds are unique in that they have a potent effect on disease development in several animal models of autoimmune/inflammatory disease without inducing suppression of adaptive immunity [7–10]. From these studies, it was obvious that the molecular target for Q compounds was novel since no known signalling pathway could explain the experimental data obtained. Furthermore, it appeared likely that the mode of action of Q compounds would be targeting early stages of immune stimulation that could be common for many autoimmune disorders while keeping the immune effector stage intact.

S100A9 [11–13] belongs to the family of calcium-binding S100 proteins and has been extensively studied [13–17]. It is expressed in granulocytes and at early stages of monocyte differentiation [14]. Complexes of S100A8 and S100A9 (S100A8/A9) are expressed and released at inflammatory sites [15,17]. A correlation between serum levels of S100A8/A9 and disease activity has been observed in many inflammatory disorders [18]. Direct inflammatory activities of the S100A8/A9 proteins include the description of mouse S100A8 as an endogenous ligand of TLR4 [17], activation of monocytes [17], and activation of endothelial cells [16,19,20]. S100A9 has also been detected on the cell surface of murine macrophages at sites of inflammation [21], but the role of surface-bound S100A9 in immunity and inflammation is still unclear. We present here data that point to a central role for S100A9 in the control of immune responses leading to inflammatory disease.

## Results

### Identifying Human S100A9 as a Candidate Target Molecule for Quinoline Carboxamides

**Figure 1:**
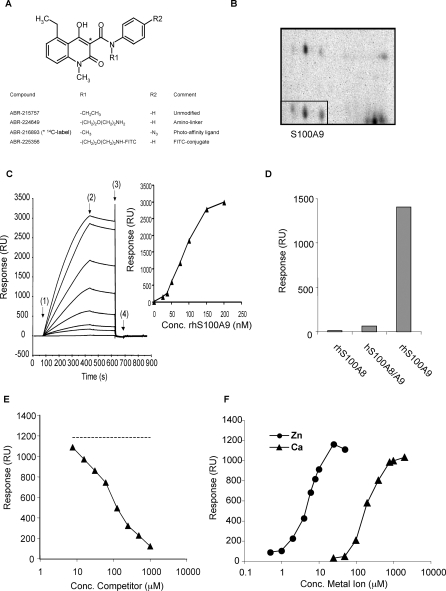
Human S100A9 Is a Target Protein for Quinolines (A) The basic structure of the quinoline compounds is shown. In the lower part of the panel, the specific modifications made in order to use these compounds as probes to isolate the target protein are shown. (B) A two-dimensional gel is shown in which the indicated spots (boxed) were subsequently identified as S100A9. The protein in all three spots was isolated separately and homogenously identified as S100A9. (C) Sensorgrams obtained after injection of 25–200 nM human S100A9 over immobilised ABR-224649 (left panel). Sensorgrams from top to bottom represent: 200, 150, 100, 75, 50, 37.5, and 25 nM S100A9 and nonspecific binding (NSB), i.e., sample buffer without S100A9. In this particular experiment, injection time was 6 min at a flow rate of 30 μl/min, and regeneration was performed with a 30-μl pulse of HBS-P buffer containing 3 mM EDTA (HBS-EP). Start injection of sample: association phase (1), running buffer: dissociation phase (2), regeneration solution (3), and running buffer again (4) constitute an analysis cycle. HBS-P with 1 mM Ca^2+^ and 10 μM Zn^2+^ was used as running and sample buffer. In the right panel, responses at steady state (after subtraction of signal in reference flow cell) were plotted versus concentration of S100A9 yielding half-maximal binding at 85 nM (*r* ^2^ = 1.00). (D) Binding of homo- and heterodimeric human S100A8 and S100A9 to immobilized ABR-224649 at a concentration of 100 nM (based on their homo- or heterodimeric molecular weight). The response at late association phase was calculated and plotted in ascending order of response magnitude. (E) Displacement of S100A9 binding to immobilised ABR-224649 by ABR-215757 is shown. S100A9 was injected for 3 min at 100 nM (i.e., at ≈*B*_max_/2 concentration) ± 7.81–1,000 μM 215757, and responses at late association were plotted against the concentration of competitor. An IC_50_ value of 37 μM was calculated in this experiment using a one-site competition model (*r* ^2^ = 1.00). The amino-linker compound, ABR-224649, showed a very similar ability to displace binding as ABR-215757 when coinjected with S100A9 over the surface (unpublished data). (F) Effect of Ca^2+^ and Zn^2+^ on binding of S100A9 to ABR-224649 is shown. S100A9, 100 nM, was injected in HBS-P buffer containing either a fixed concentration of Ca^2+^ (1 mM) or Zn^2+^ (10 μM), with Zn^2+^ and Ca^2+^ concentrations titrated from 0–50 μM and 0–2,000 μM, respectively. Responses at late association phase were plotted versus metal ion concentration, and EC_50_ values of 5.5 μM for Zn^2+^ and 193 μM for Ca^2+^ were calculated using a sigmoidal dose-response model.

In order to identify the target molecule of Q compounds, we synthesised analogs of these compounds containing linkers that would facilitate detection of the interaction between these molecules and protein targets (Figure 1A). The molecule was modified as indicated in the R1 and R2 position to create a compound suitable for photoaffinity labelling of proteins (ABR-216893; the asterisk [*] indicates a ^14^C atom in this compound), or in the R1 position to create a compound (ABR-225356) labelled with FITC. Since no reliable in vitro system has been established for assaying the biological effect of Q compounds, we verified that these linker-containing Q compounds still had biological effect using the in vivo model acute experimental autoimmune encephalomyelitis (aEAE) (unpublished data). We also used the FITC-labelled compound (ABR-225356) to investigate binding to human peripheral blood mononuclear cells (PBMC). We observed that only the monocyte (CD14^+^) fraction was surface stained with ABR-225356 (unpublished data). On the basis of this, we decided to use human peripheral blood monocytes as a source of protein in our effort to isolate quinoline-binding molecules.

Human PBMC were separated into CD14^+^ and CD14^−^ fractions, incubated with ^14^C labelled ABR-216893, and photoaffinity labelled. The membrane fraction of both cell populations was subsequently prepared, and the proteins separated on two-dimensional gels followed by autoradiography. Labelled proteins found exclusively on gels from the CD14^+^ cell fraction were extracted from the gels and identified using MALDI-TOF (matrix assisted laser desorption/ionization time-of-flight). The most prominent binding protein was identified as S100A9 and was selected for further analysis (Figure 1B). In the next step, recombinant human S100A9 was analysed for binding to a defined Q compound (ABR-215757; currently in clinical development for treatment of SLE) using surface plasmon resonance (SPR). As shown in Figure 1C, it was evident that recombinant S100A9 bound strongly to ABR-215757 coupled to solid phase. As S100A9, in most cases, is found colocalised with S100A8 at inflammatory sites, we decided to analyze homo- and heterodimeric complexes of S100A8 and S100A9 for their interaction with Q compounds. Figure 1D shows that binding was more or less exclusively restricted to homodimeric S100A9, whereas only weak binding was observed for the S100A8/A9 complex, and close to baseline levels for S100A8. Last, we determined that the Q compound/S100A9 interaction could be competed in a dose-dependent way by free compound (Figure 1E). Additional proteins were identified using photoaffinity labelling but could not be verified using follow-up SPR analysis. We concluded from these studies that human S100A9 was a potential pharmacological target for Q compounds.

S100A9 belongs to the S100 family of proteins that are known to be Ca^++^ binding proteins and are involved in inflammatory processes [18]. However, S100A9 has also been shown to bind Zn^++^, and this interaction might have conformational consequences for the protein [22]. We therefore investigated the dependence of the interaction between ABR-215757 and S100A9 for both these divalent ions. When Ca^++^ or Zn^++^ were titrated in the presence of a fixed concentration of either Zn^++^ or Ca^++^, we found that both ions were required for S100A9 binding to Q compounds (Figure 1F). If titration was carried out in the absence of either Ca^++^ or Zn^++^, the binding of S100A9 to ABR-215757 was reduced to baseline values (Figure S5). It should also be noted that the levels of Ca^++^ or Zn^++^ required for optimal S100A9/quinoline interaction are within the concentration range found for these ions in human serum [23]. We are aware of the fact that S100A9 is prone to form dimers [16,18] and complexes of even higher oligomeric states at high concentrations, and therefore expect the interaction between Q compounds and human S100A9 to be occurring primarily with at least bivalent structures of the molecule. This assumption was supported by the complex kinetics and sigmoidal-shaped dose-response curve for S100A9 binding to immobilised Q compound.

### Q compounds Inhibit the Zn^++^-Dependent Interaction between S100A9 and RAGE

**Figure 2:**
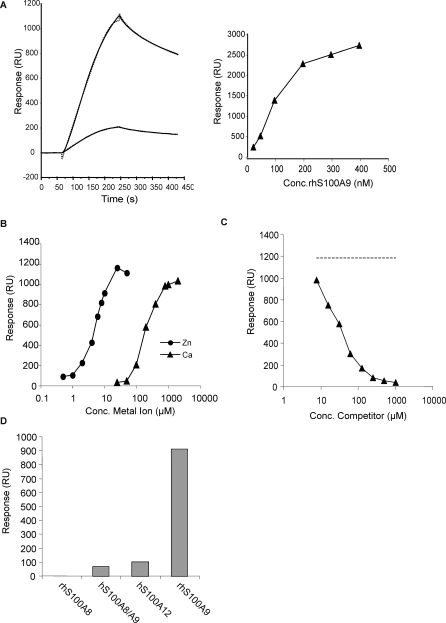
Human S100A9 Binds Human RAGE (A) Left: sensorgrams showing association and dissociation phase after injection of 100 nM hS100A9 (upper) and hS100A8/9 (lower) over RAGE immobilized at a density of approximately 3,000 RU (solid lines). Curves were fit to a 1:1 binding model with mass transfer (dotted line) and yielded K_D_ values of 38 and 9.4 nM with maximum responses at 1,200 and 220 RU for homodimeric and heterodimeric hS100A9, respectively. The somewhat lower affinity for homodimeric S100A9 is due to a higher off-rate. Right: responses at late association phase plotted against the concentration (Conc.) of S100A9 showing half-maximal binding at approximately 100 nM. (B) Zn^2+^ and Ca^2+^ concentrations were titrated as described in Figure 1F. EC_50_ values of 7.1 μM for Zn^2+^ and 146 μM for Ca^2+^ were calculated using a sigmoidal dose-response model. (C) Displacement curve showing inhibition of 100 nM S100A9 binding to the RAGE surface with 7.81–1,000 μM ABR-215757 as competitor. Competitor in the absence of S100A9 showed background or minimal direct binding to RAGE (unpublished data). In this experiment, the resulting displacement curve yielded 50% inhibition at 26 μM. Dashed line indicates signal for S100A9 in the absence of competitor. (D) Binding of 100 nM S100 proteins (injected for 3 min at 30 μl/min) to immobilised RAGE in the presence of 10 μM Zn^2+^ and 1 mM Ca^2+^ with responses calculated from sensorgrams at late association phase. Comp, competitor.

The next question was to gain some understanding concerning the mechanism whereby S100A9 binding by Q compounds could inhibit autoimmune disease. Members of the S100 protein family have been shown to interact with the proinflammatory molecule RAGE (receptor for advanced glycation end products) [18,24], but to our knowledge, there are no data in the literature showing a direct, physical interaction between RAGE and S100A9. Furthermore, it has been shown that soluble RAGE may alleviate aEAE [25]. We therefore decided to investigate whether human S100A9 was a human RAGE ligand using SPR. In this study, RAGE was covalently coupled to the sensor chip to allow exposure of the extracellular domain of RAGE to S100A9, thus reconstituting a biological model in which anchored membrane receptor interacts with soluble ligands. As shown in Figure 2A, S100A9 interacted strongly with immobilised RAGE when injected at the concentration yielding half-maximal binding to ABR-224649, almost a 6-fold higher response compared to the S100A8/A9 heterodimer. Moreover, the binding of S100A8 to RAGE was negligible. The interaction between S100A9 and RAGE was also strictly dependent on the presence of physiological concentrations of both Ca^++^ and Zn^++^ (Figure 2B). Given the similarities between the binding conditions for S100A9 interaction with RAGE and Q compounds, we proceeded to test whether a Q compound could compete for RAGE binding to S100A9. Indeed, ABR-215757 in increasing concentrations competed for RAGE-S100A9 binding in the presence of Ca^++^ and Zn^++^ (Figure 2C). Furthermore, direct binding of ABR-215757 to RAGE was not observed, indicating that the inhibition of the interaction was mediated by binding of ABR-215757 to S100A9 (unpublished data). In a separate experiment in which human RAGE/Fc and Fc alone were allowed to interact with S100A9 immobilised on the chip, we observed no interaction with Fc alone (Figure S4A). Thus, under our standard conditions, homodimeric S100A9 is the primary RAGE ligand (Figure 2D), as well as target for Q compound binding.

### Q Compounds Inhibit the Zn^++^-Dependent Interaction between S100A9 and TLR4

**Figure 3:**
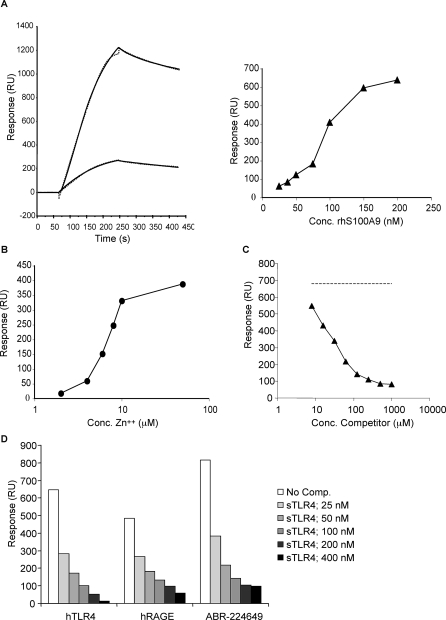
Interaction of Human S100A9 with Human TLR4/MD2 (A) Left: binding of 100 nM S100A9 (upper) and S100A8/9 (lower) to immobilized TLR4/MD2 (coupling density ∼3,000 RU) represented as sensorgrams (solid lines). Best fit of curves was obtained with 1:1 model with mass transfer (dotted lines) with K_D_ values of 2.1 and 3.8 nM and maximum responses at 1,270 and 280 RU, respectively. Right: half-maximal binding at approximately 77 nM was obtained when plotting responses at late association phase against concentration (Conc.) of S100A9. (B) Influence of Zn^2+^ on S100A9 binding to hTLR4/MD-2 at a fixed concentration of Ca^2+^ (1 mM). Half-maximal binding was obtained at 6.4 μM Zn^2+^. (C) Displacement curve showing inhibition of S100A9 binding to immobilised hTLR4/MD2 at 100 nM with the competitor (ABR-215757) added in a concentration range from 7.81 to 1,000 μM. Direct binding of ABR-215757 to hTLR4/MD2 was negligible (unpublished data). In this experiment, an IC_50_ value of 23 μM (*r* ^2^ = 0.998) was calculated after fit of data to a one-site competition model. Response for S100A9 in the absence of compound is indicated by the dashed line. (D) Blocking of human S100A9 binding to immobilized TLR4/MD2, RAGE and Q compound by human TLR4/MD2 complex in solution. Comp, competitor.

Having defined S100A9 as a RAGE ligand, we wanted to investigate whether other proinflammatory signalling molecules would also interact specifically with human S100A9. We had noted that one Q compound had been shown to inhibit lipopolysaccharide (LPS)-induced toxic shock [26]. We therefore decided to investigate whether TLR4 could be a possible S100A9 ligand and whether Q compounds could interfere with such interactions. Since TLR4 is inactive in the absence of the coreceptor MD2, we here used a complex of human TLR4 and MD2 for amine coupling to a biosensor chip to be used in a SPR assay. As shown in the left panel of Figure 3A, S100A9 showed strong binding when injected over immobilised TLR4/MD2 using our standard conditions, and produced a more than 5-fold higher signal than the S100A8/A9 heterodimer. Furthermore, the signal obtained after injection of S100A9 was proportional to the amount of TLR4/MD2 coupled to the solid phase (Figure S7). We could also demonstrate that the binding of S100A9 to TLR4/MD2 is TLR4 specific since the TLR4/MD2 complex interacted with immobilised S100A9 with high affinity whereas MD2 alone did not (Figure S4B). Hence, we can show here for the first time that human S100A9 is an endogenous TLR4 ligand. The interaction between human S100A9 and TLR4/MD2 was strictly dependent on the presence of both Zn^++^ (Figure 3B) and Ca^++^ (unpublished data), which could explain why this interaction has not been previously described. We then proceeded to investigate whether the Q compound ABR-215757 could interfere with human S100A9 binding to TLR4/MD2. As shown in Figure 3C, a dose-dependent inhibition of the interaction was observed, whereas only very weak inhibition was seen with a control substance (Figure S6). We also wanted to test whether soluble TLR4/MD2 could displace binding of S100A9 to immobilised TLR4/MD2.

Interestingly, TLR4/MD2, injected together with S100A9, was not only able to efficiently block the interaction of S100A9 with immobilised TLR4, but also inhibit the interaction between S100A9 and immobilised Q compound and RAGE, respectively (Figure 3D). This observation indicates that TLR4/MD2, RAGE, and Q compound compete for the same binding region in human S100A9. The TLR4/MD2 complex is known to bind LPS, and therefore we investigated whether LPS could interfere with the binding of human S100A9 to the immobilised human TLR4/MD2 receptor complex. In contrast to the dose-dependent displacement of S100A9 binding by soluble TLR4/MD2, LPS had no effect on this interaction even at 200 ng/ml (Figure S1A). Thus, human S100A9 in the presence of Ca^++^ and Zn^++^ can interact specifically with two distinct proinflammatory receptors.

### Mouse S100A9 Interacts with Q Compounds, Mouse RAGE, and Mouse TLR4/MD2

**Figure 4:**
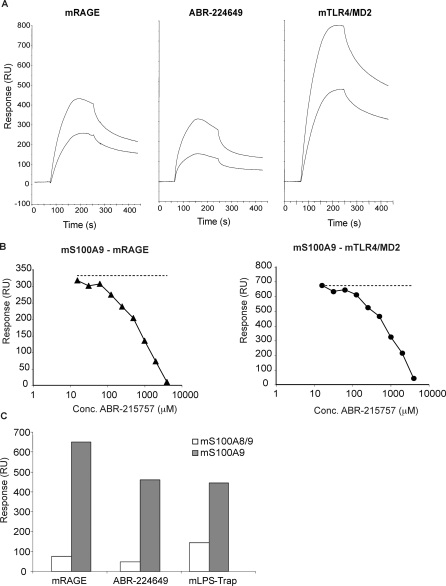
Analysis of Mouse S100A9 Interaction with Mouse RAGE, Q Compound, and Mouse TLR4/MD2 (A) Sensorgrams generated after injection of 50 and 100 nM mouse S100A9 (3 min at 30 μl/min) over indicated ligands coupled to the sensor chip. (B) Inhibition curves for displacement of 100 nM mouse S100A9 interacting with mouse RAGE and mLPS-Trap, respectively, using ABR-215757 as competitor. Conc, concentration. (C) Binding of 100 nM mouse S100A9 homodimers and S100A8/A9 heterodimers to the indicated surfaces.

With the results above, we had a foundation on which to understand the effect of Q compounds on inflammatory disease in humans. However, these compounds have also shown a broad activity in several disease models in mice [1,8–10]. Thus, we needed to validate our findings using mouse proteins. Figure 4A illustrates that very similar results to those obtained with the human proteins were obtained both with regard to mouse S100A9 binding to mouse RAGE, mouse S100A9 binding to Q compound, and mouse S100A9 binding to the mouse TLR4/MD2 fusion protein (mLPS-Trap [27]). Also, all interactions showed similar requirements for Ca^++^ and Zn^++^ (unpublished data). Furthermore, the interaction of mouse S100A9 with solid-phase mouse RAGE and mouse TLR4/MD2 could both be competed by the Q compound ABR-215757 (Figure 4B). Analogous to the human S100A9-TLR4/MD2 interaction, soluble TLR4/MD2 displaced mS100A9 binding to immobilised TLR4/MD2 in a manner independent of both LPS and MD2 (Figure S1B). Moreover, as was shown for human S100A9, homodimeric mouse S100A9 bound much stronger to immobilised Q compound, RAGE and TLR4 than as a heterodimer with S100A8 (Figure 4C). We conclude from this series of experiments that neither the interactions between S100A9 with RAGE and TLR4/MD2, nor the competition of this interaction by Q compound, are species specific.

### Q Compound Binding to S100A9 Shows a Relationship with Pharmacological Activity

Having determined that S100A9 interacted specifically with Q compounds, we next wanted to determine whether S100A9 would qualify as a bona fide pharmacological target for the Q compounds. To this end, we selected six compounds (see Table S1) from our chemical libraries of Q compounds [28] and tested these for their binding strength to human and mouse S100A9 and to human and mouse S100A8/A9 heterodimers, their potency in inhibiting the interaction between human and mouse S100A9 and RAGE, and their potency in inhibiting the interaction between human and mouse S100A9 and TLR4/MD2.

**Figure 5:**
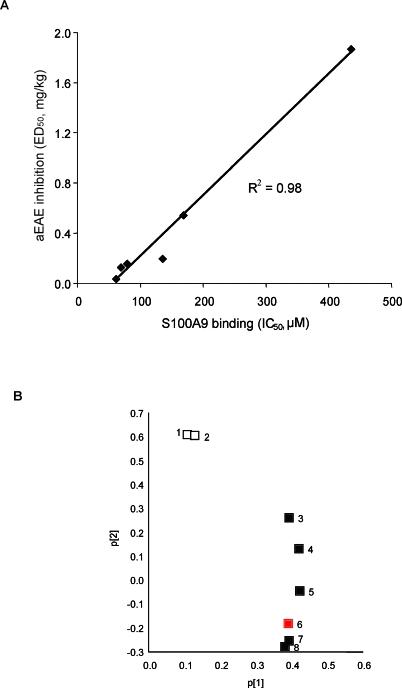
PCA Loading Plot of the Relationship between Q Compound S100A9 or S100A8/A9 Binding Activity and aEAE Inhibition (A) Correlation between the binding strength to S100A9 of the Q compounds (Table S1) and their efficacy in inhibiting aEAE in vivo. (B) Loading plot of the first two principal components (p[1] and p[2]) demonstrating the relationship between S100A9 and S100A8/A9 binding activity and aEAE inhibition. 1: mS100A8/A9 and mRAGE; 2: mS100A8/A9 and mLPS-Trap; 3: mS100A9 and ABR-224649; 4: mS100A9 and mLPS-Trap; 5: mS100A9 and mRAGE/hFc; 6: aEAE; 7: hS100A9 and rhTLR4/MD-2; and 8: hS100A9 and rhRAGE/Fc.

Multivariate analytical tools (principal component analysis [PCA] and partial least squares projections to latent structures [PLS]) were used to derive the structure-activity relationship (SAR) for the binding activity of a series of quinoline compounds to the S100A9 homodimers and the S100A8/A9 heterodimers (Table S1). When the potency of these compounds in inhibiting aEAE in vivo was directly correlated to their potency in inhibiting the interaction between human S100A9 and human RAGE, an excellent correlation was observed (*R* ^2^ = 0.98) (Figure 5A).

We then proceeded to apply PCA modelling to the dataset, i.e., the five S100A9 and the two S100A8/A9 assays and the aEAE model, a two-component model with *R^2^X* = 0.97 and *Q^2^* = 0.87, was obtained. The first model dimension reflected as much as 68% of the total variation. The principal component (PC) scores revealed differences between the homodimer and the heterodimer. An overall inspection of the loading plot (Figure 5B) reveals that the aEAE inhibition (point 6) and the S100A9 homodimer binding (points 3–5, 7, and 8) are situated close to each other on the first principal component (p[1]), indicating that strong positive correlations exist among them. On the other hand, the S100A8/A9 heterodimer binding (points 1 and 2) are more distant from the aEAE point, meaning that S100A8/A9 heterodimer binding and aEAE are not strongly correlated.

A set of five quinoline derivatives incorporating different substitution patterns at position 5 with relative binding affinities measured to the S100A9 homodimer in the mS100A9–RAGE interaction assay was used to derive the SAR of the binding activity of the quinoline derivatives towards the S100A9 homodimers. The results confirmed that the structural modifications carried out on the 5-position have a profound effect on binding affinity. The PLS evaluation resulted in a three-component model, obtained with cross validation, giving a SAR model with *R* ^2^ *Y* = 0.99 (85% + 12% + 2%) and *Q* ^2^ = 0.81, which indicates that mS100A9 homodimer binds the quinoline compounds with a high structural selectivity. The observed and predicted half-maximal inhibitory concentration (IC_50_) values for these compounds for inhibition of mS100A9/RAGE interactions are shown graphically in Figure S2 and very similar results were obtained for all other S100A9 interactions investigated. The analysis pointed out the major importance of steric and hydrophobic factors (*L*, *B_1_*, π of the 5-substituent, and the acid strength of the 4-hydroxy group. Furthermore, local electrostatics at positions 4 and 5 were important for the biological activity. In an ensuing step, the SAR model was further tested using an additional quinoline derivative, i.e., ABR-212662 (Table S1). This compound was selected based upon its substitution and variation within the activity range, i.e., being unsubstituted in the 5-position and displaying low binding activity. The observed and predicted binding activities for this compound showed high correspondence and were 1,026 and 1,235 μM, respectively. Hence, this SAR model is robust and valid for prediction as used. We conclude from the data shown that S100A9 by its SAR to disease inhibition qualify as a pharmacological target molecule for Q compounds.

### In Vivo Validation of S100A9 as a Pharmacological Target

Having shown that S100A9 binding by Q compounds showed a SAR with their activity in inhibiting autoimmune disease, the next step in our investigation was to validate S100A9 as a drug target in vivo. We first considered the obvious experiment of using S100A9 null mice [29]. To this end, we back-crossed the S100A9^−/−^ animals against C57BL/6 mice and induced experimental autoimmune encephalomyelitis (EAE) using MOG peptide (Figure S8). We observed that the S100A9^−/−^ animals had a more severe disease than C57BL/6 controls, but still responded to treatment with Q compounds. This was an unexpected result given the very strong SAR between the binding strength of Q compounds to S100A9 and their potency in inhibiting aEAE (Figure 5). However, the absence of an obvious functional phenotype with a specific gene deletion does not necessarily prove that the protein it codes for has an insignificant function in an intact host. The S100 family of proteins is large and complex. For example, whereas S100A12 has been shown to be a RAGE ligand in humans [30], its gene is absent in the mouse genome [31]. S100A8 is expressed almost exclusively as a S100A8/A9 heterodimer, but whereas S100A9^−/−^ mice are viable, the S100A8^−/−^ genotype is embryonically lethal [32]. In addition, S100A9^−/−^ mice show spontaneous alterations of their inflammatory response also in other experimental models [17]. Given that S100A9 convey important biological functions, it can be suspected that biological redundancy may occur in the S100A9^−/−^ animal, in which another molecule(s), maybe from the S100 family, would serve as a ligand for RAGE, TLR4, and Q compounds. Such a molecule could have very limited function in a genetically intact animal.

To be able to perform the in vivo validation of S100A9 as a pharmacological target for Q compounds in wild-type animals, we therefore turned to an alternative approach. Hence, we decided to generate a set of monoclonal antibodies to S100A9 that could compete for S100A9 binding to RAGE and TLR4/MD2. S100 proteins are rather conserved during evolution [33,34]. Assuming that their biological function also has been conserved, one may speculate that it would be difficult to obtain antibodies to key regulatory epitopes using xenoimmunisation. We therefore elected to immunize S100A9^−/−^ mice with recombinant human S100A9 in order to obtain antibodies to novel, potentially functional, epitopes on the S100A9 protein. Approximately 50 S100A9-specific hybridomas were obtained in this experiment, and one of these, 43/8, was used for further validation.

**Figure 6:**
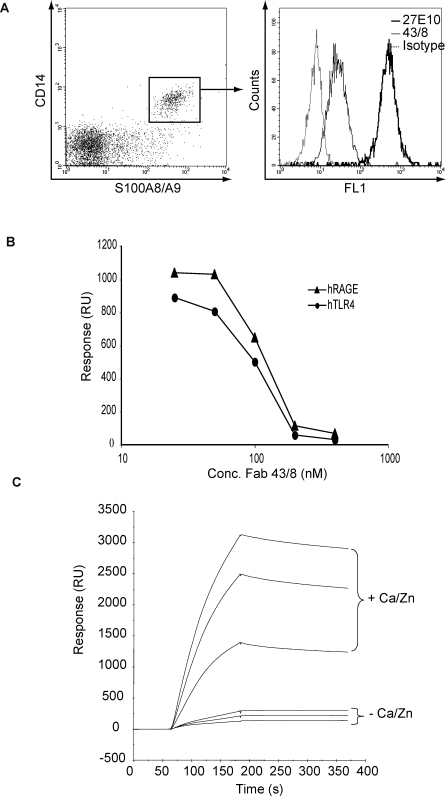
Fab 43/8 Blocks Binding of S100A9 to RAGE and TLR4 (A) FACS analysis of human PBL using 43/8, the S100A8/A9 specific antibody 27E10 and isotype control. FL1, fluorescence channel 1 (green). (B) Inhibition curves for binding of 100 nM human S100A9 to immobilised human RAGE and human TLR4/MD2 in the absence or presence of 25 to 400 nM Fab 43/8. After preincubation of S100A9 ± Fab for 1 h at room temperature in HBS-P buffer containing 1 mM Ca^2+^ and 10 μM Zn^2+^, samples were injected for 3 min at 30 μl/min. Note: molar ratio homodimeric S100A9/monovalent Fab is 8:1, 4:1, 2:1, 1:1, and 0.5:1 at the various Fab concentrations. Conc, concentration. (C) Influence of Ca^2+^ and Zn^2+^ on binding of human S100A9 to amine-coupled intact 43/8 antibody (coupling density ∼5,000 RU). Sensorgrams generated after injection of S100A9 at 100, 200, and 400 nM (3 min at 30 μl/min) over 43/8 in the absence (−Ca/Zn) or presence (+Ca/Zn) of 1 mM Ca^2+^ and 10 μM Zn^2+^.

Figure 6 shows the basic features of the 43/8 antibody. It binds both human and mouse S100A9 (Figure S3A). The antibody will also surface stain human monocytes in fluorescence-activated cell sorting (FACS) analysis but not as brightly as the S100A8/A9-specific antibody 27E10 (Figure 6A). Fab fragments of the 43/8 antibody (Figure S3B) will also inhibit the interaction of S100A9 and RAGE, as well as S100A9 and TLR4/MD2, showing almost complete inhibition at a concentration of 200 nM (Figure 6B). Very similar results were obtained with intact 43/8 antibody, but not with an isotype control (unpublished data). We could also demonstrate that the epitope recognized by the 43/8 antibody is exposed in an optimal way only in the presence of Ca^++^ and Zn^++^. As is shown in Figure 6C, human S100A9 binds to immobilised intact 43/8 antibody with a more than 10-fold higher signal when injected with Ca^++^ and Zn^++^.

**Figure 7:**
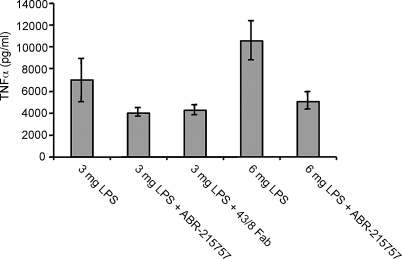
Inhibition of TNFα Induction by 43/8 Fab and ABR-215757 C57BL/6 mice were treated with PBS, 43/8 Fab, or ABR-215757 for 2 h before being challenged with 3 or 6 μg of LPS intraperitoneally, as indicated. Two hours after LPS challenge, the animals were sacrificed, and the level of TNFα in serum was determined. Error bar indicates standard error of the mean (SEM).

Vogl et al. [17] has demonstrated that the induction of systemic TNFα production by LPS is perturbed in S100A9^−/−^ animals. We therefore selected this model for our in vivo validation. C57BL/6 mice were treated with the Q compound ABR-215757 2 h before being challenged intraperitoneally with 3 or 6 μg of LPS. Ninety minutes later, the animals were sacrificed, and the serum TNFα levels were determined. As shown in Figure 7, Q compound significantly inhibited TNFα production at both levels of LPS challenge, with the effect being most pronounced using the 6-μg challenge. We then proceeded to use the 43/8 Fab in the same assay. As shown in Figure 7, after challenge with 3 μg of LPS, the TNFα production was significantly inhibited also using 43/8 Fab. We conclude from this experiment that Q compounds, or an antibody Fab fragment that mimics Q compounds in the sense that it inhibits S100A9 interaction with TLR4 and RAGE, can inhibit a biological activity shown to be compromised in S100A9^−/−^ animals [17]. Hence, we consider these data as an in vivo validation of S100A9 as a pharmacological target for Q compounds.

## Discussion

Although the prognosis and clinical management of patients with chronic inflammatory diseases has improved during the last decades, there is still a great medical need for new treatments. Treatments especially that do not compromise immune function and that are suitable for chronic dosing are urgently needed. A group of compounds that fulfils these criteria are the Q compounds that have been in clinical development for over two decades, but whose molecular target and mode of action are unknown. The present investigation defines S100A9 as one molecular target for Q compounds, and their detailed effects on autoimmune disease in mice and humans [1–5,7–10] can now be studied in a more rational fashion. Interestingly, the effect of Q compounds resembles the phenotype recently described for the S100A9 knockout model, in that a diminished TNFα response after LPS challenge was observed [17]. That the target molecule for these compounds, S100A9, interacts with signalling pathways that are early and potent mediators of proinflammatory responses (RAGE and TLR4) could shed some light on the ability of Q compounds to mediate this effect without causing overt suppression of adaptive immunity. It can be speculated that the interference with proinflammatory signalling at the level of antigen-presenting cells may suppress the reactivity of autoimmune T cells. In addition, human S100A9 can now be regarded as a novel therapeutic target for the treatment of autoimmune diseases.

The interactions between S100A9 and Q compound, RAGE, or TLR4/MD2 were all strictly dependent on physiological levels of Ca^++^ and Zn^++^. The interaction between S100A9 and Zn^++^ especially appears to induce a dramatic structural change in the protein, which also was shown to significantly affect the binding of the 43/8 monoclonal antibody. It is interesting to note that Zn^++^ has been shown to have a profound impact on the structure of other S100 proteins [35]. Also, elevated levels of Zn^++^ are seen at inflammatory sites, and many extracellular proteins contain Zn^++^ binding sites [23]. Thus, it can be speculated that the elevation of Zn^++^ is a feedforward signal for inflammation and acts by inducing conformational changes in proteins and thereby facilitating novel interactions. That both RAGE and TLR4/MD2 interact with the same surface on human S100A9 and are competed by Q compounds is intriguing. We have investigated several of the mouse and human TLRs for binding to the same interphase on human S100A9 without finding additional targets (unpublished data). However, we expect that other proinflammatory molecules will eventually be shown to interact with the same molecular surface on human S100A9, and are also open for the idea that other forms of S100 protein combinations may bind to proinflammatory mediators. The common theme remains that a molecular form of S100 proteins can interact with several proinflammatory mediators as a mechanism to modulate the quality of the immune response and inflammatory reactions.

At first glance, the data presented here are in conflict with previously published data [17]. In this study, biosensor experiments were conducted with recombinant murine S100A8 immobilized on the chip using amine coupling. Binding to murine TLR4/MD2 fusion protein (mLPS-Trap) was demonstrated, as was the ability of murine S100A8, but not S100A9, to stimulate TNFα production of bone marrow cells from wild-type mice. In the present study, however, S100 proteins were injected over a surface with immobilized TLR4 to preserve the Ca/Zn conformation of S100A9 that is a prerequisite for binding activity. Under these conditions, S100A8 only showed weak interaction with TLR4. The biological reason for this discrepancy may be explained by the fact that the biological function of S100A8 and S100A9 is regulated in a complex manner, which additionally may differ between mice and men. For example, human S100A9 activates integrin affinity of CD11b/CD18 on monocytes, whereas human S100A8 has no effect [36]. Vice versa, murine S100A8 activated murine macrophages whereas murine S100A9 was inactive. Regulatory effects of human S100A9 on tubulin metabolism are completely abrogated by phosphorylation of S100A9 at threonine 113 [37]. This MAPK p38-dependent phosphorylation site is not conserved in murine S100A9. It seems therefore likely that murine S100A9 may mediate so far undefined regulatory mechanisms in vivo, which may be responsible for the discrepancy between different experimental findings between mice and men in vivo and in vitro. Activation of the innate immune system is crucial for initiation and amplification of many inflammatory responses and autoimmune diseases. During this process, endogenous danger signals called alarmins or damage-associated molecular patterns (DAMPs) play a pivotal role via interaction with specific pattern-recognition receptors [38]. S100A8 and A9 have been identified as important endogenous DAMPs due to their activation of TLR4 [14,17]. Thus, specific blocking of S100 proteins, as presented here, represents the first report of targeted intervention with a DAMP-mediated inflammatory process, which has already shown pharmacological activity in mice and men [4,10].

S100A8 and S100A9 are two members of the S100 protein family. Multivariate analytical tools were used to derive the SAR for the binding activity of a series of Q compounds towards the S100A9 homodimer and the S100A8/9 heterodimer, with the assumption that similar analogs bind to the same binding site in a similar binding mode. The results indicate that the Q compounds bind the S100A9 homodimers with high structural selectivity and that this binding showed a strong correlation to their ability to inhibit autoimmune disease. On the other hand, the correlation between Q compound binding to S100A8/A9 heterodimers and inhibition of autoimmune disease was poor. The bulk of S100A8 and S100A9 protein is expressed as S100A8/A9 heterodimers and most of this protein is found as soluble protein in serum. S100A8 and S100A9 are also expressed on the cell surface of monocytes [12,15]. Whether the pharmacological activity of Q compounds is primarily mediated by blocking soluble or membrane-bound S100A9 will be a subject for future studies.

## Materials and Methods

### Reagents.

Murine and human S100A8, S100A9, S100A8/A9, and human S100A12 were either produced recombinantly in Escherichia coli or purified from granulocytes; essentially as described [39]. Mouse TLR4/MD2 fusion protein (mLPS-Trap) was obtained through collaboration [27]. Carrier-free recombinant human RAGE/Fc (hRAGE), human IgG1Fc (hFc) mouse RAGE/Fc (mRAGE), human TLR4/MD-2 complex (hTLR4/MD-2), human MD-2, mouse anti-hRAGE (clone #176902), mouse anti-hTLR4 (clone #285219), and rat anti-mTLR4 (clone #267518) were purchased from R&D Systems. The mouse anti-human S100A9 monoclonal antibody (clone 43/8) was produced in-house by immunisation of S100A9^−/−^ mice. LPS from E. coli was obtained from Sigma. Protein concentration was determined using the microtiter plate BCA assay from Pierce with bovine serum albumin as standard, or by absorbance measurement at 280 nm using molar absorption coefficient. Biotinylation of the 43/8 monoclonal antibody was made using the NHS-LC-biotin reagent from Pierce. The antigen binding fragment (Fab) of mouse anti-human S100A9 monoclonal antibody 43/8 was prepared by enzymatic digestion on immobilized ficin using the mouse IgG1 Fab preparation kit from Pierce. Gel electrophoresis under denaturing conditions was run on 4%–12% Bis-Tris NuPAGE gels with MES-SDS as running buffer (Invitrogen). For details on the synthesis and features of quinoline compounds, see Jönsson et al. [28] and references therein.

### FACS analysis and protein identification.

The following antibodies and second steps reagents were used for surface stain of human PBMC, CD14-APC, mouse IgG1 (BD Biosciences Pharmingen), 27E10-FITC (BMA Biomedicals), Streptavidin-Alexa Fluor 488 (Invitrogen), and biotinylated 43/8 monoclonal antibody. Stained cells were analyzed by flow cytometry on a FACSCalibur (Becton Dickinson) using CellQuest software. For protein isolation, PBMC were divided into CD14^+^ and CD14^−^ populations using positive selection of CD14^+^ cells with magnetic beads (Miltenyi Biotec). Both cell types were incubated with ABR-216893 in the dark on ice after which the cells were exposed for a light source for 30 min, lysed, and protein extracts prepared as described [40]. The proteins were subsequently subjected to conventional two-dimensional gel analysis and autoradiography. Radioactive spots that were present selectively in extracts from CD14^+^ cells were isolated, the proteins eluted, trypsin digested, and prepared for analysis in a Bruker Reflex III instrument (Bruker Daltonik) using protocols and software supplied by the manufacturer.

### In vivo experiments.

TNFα induction after intraperitoneal challenge with LPS was performed essentially as described [17]. In brief, mice were pretreated for 2 h with 10 mg/kg ABR-215757 or PBS, after which 3 or 6 μg of LPS was injected intraperitoneally. After an additional 90 min, the animals were sacrificed, and the level of TNFα in blood was determined using commercial TNFα antibodies (eBioscience; http://www.ebioscience.com).

### Anti-S100A9 monoclonal antibodies.

S100A9^−/−^ mice (10 wk of age) were injected intraperitoneally with 100 μg of recombinant human S100A9 precipitated in alum. Six weeks later, the mice were boosted with the same dose of antigen and the spleen cells fused to SP2/0 5 d later. S100A9 reactive clones were selected using ELISA, and positive clones were subcloned by limiting dilution.

### Surface plasmon resonance.

The SPR analysis was carried out with the Biacore 3000 system (GE Healthcare). Sensor chips, amine coupling kit, immobilization and running buffers, and regeneration solutions were obtained from GE Healthcare. Working solutions of all reagents used for Biacore analysis were prepared in 0.01 M Hepes, 0.15 M NaCl (pH 7.4) containing 0.005% v/v Surfactant P-20 (HBS-P; GE Healthcare) by buffer exchange on Fast Protein Desalting Micro-Spin Columns from Pierce. ABR-215757 was immobilised onto a CM5 chip through an amino-linker (ABR-224649). Other reagents (i.e., RAGE, TLR4/MD2, and various antibodies) were immobilised to the aimed density using random amine coupling chemistry. Activity of ligands after immobilisation was tested by injecting specific antibodies (unpublished data). In some experiments, S100A9 was immobilized either by random amine coupling or with a known orientation (i.e., by sulhydryl group conjugation to the only cysteine in S100A9 at position 3).

Binding to the various surfaces was performed by injecting the analyte at a flow rate of 30 μl/min in a physiological buffer (HBS-P) containing 1 mM Ca^2+^ and 10 μM Zn^2+^ as proposed for S100A8/9 by Robinson et al. [16]. A typical analysis cycle consists of: (1) pumping running buffer for 1 min to obtain a stable baseline; (2) injection of sample for an appropriate period of time (association); (3) pumping running buffer for 2.5 min (dissociation); (4) injection of a short pulse (30 s) of 15 μl 10 mM glycine-HCl (pH 2.0) (regeneration); and (5) pumping running buffer for 2 min (stabilisation after regeneration) at a flow rate of 30 μl/min. As S100A9 is a calcium-binding protein and shown to require low concentrations of Zn^2+^ to adapt a biologically active conformation [16], titration of Zn^2+^ and Ca^2+^ for optimal binding of S100A9 to immobilised ABR-224649, RAGE, and hTLR4/MD2 was performed. In one experiment, S100A9 was injected into a buffer with a fixed Ca^2+^ concentration (1 mM) and Zn^2+^ in the range 0–50 μM. In a second experiment, Ca^2+^ was varied from 0–2 mM at a fixed Zn^2+^ concentration (10 μM). In subsequent analyses, regeneration was carried out under more mild conditions, i.e., by injecting 30 μl of HBS-P containing 3 mM EDTA (HBS-EP; GE Healthcare) for 60 s, to prolong the lifespan of the chip.

In order to study displacement of S100A9 binding to immobilised Q compound, RAGE, and TLR4/MD2, S100A9 at a concentration yielding approximately half-maximal binding was incubated in the absence or presence of serially diluted Q compounds. Compounds were also injected over the immobilised surfaces in the absence of S100A9 to exclude, or make possible correction for, any direct binding of compound to the surface.

Evaluation was carried out using BIAevaluation Software version 3.2 (GE Healthcare). The response at steady-state was obtained by fit of sensorgrams to standard binding models, where appropriate, or calculated as responses at late association or early dissociation phase using the Steady state affinity function in BIAevaluation. Affinity was determined from kinetic analysis (on- and off-rates) or as the apparent affinity after plotting responses versus concentration of analyte in a saturation curve. In the inhibition assay format, the competitor concentration yielding 50% inhibition (IC_50_) was calculated by fitting data to a one-site competition model in GraphPad Prism.

### SAR analysis.

Multivariate analytical tools (PCA and PLS) were used to derive the SAR for the binding activity of a series of quinoline compounds to the S100A9 homodimer and the S100A8/A9 heterodimer (Table S1). The software SIMCA-P+ 11 (Umetrics AB; http://www.umetrics.com) was used to conduct the multivariate data analysis. A number of physicochemical descriptors for size, lipophilicity, and electronic characteristics were used to correlate structural or property descriptors of the compounds with their biological activities. The 5-substituents of the quinoline compounds were described by two dimensionally–based structure descriptors, i.e., STERIMOL parameters (*L*, *B_1_*, and *B_5_*) as steric parameters, and the substituent constant π as a hydrophobic parameter. The experimentally determined and assigned carbon ^13^C-NMR chemical shifts of atoms from positions 3 to 10 on the quinoline template were used to reflect local electrostatics. There were only minor ^13^C-NMR shift differences between carbons in positions 2, 11, and 1′-4′, and the ^13^C shifts of these latter atoms were not used when establishing the SAR models. The acidity constants (pKa) in water of the corresponding *ortho*-substituted benzoic acid derivatives were used to correlate structure and acid strength of the 4-hydroxy group. The steric and hydrophobic parameters used were 2D-based structure descriptors known from the literature [41]. ^13^C NMR spectra were recorded with an operating frequency of 125.8 MHz. Spectra were recorded in D_2_O with a small addition of NaOD at ambient temperature. The shift scale was referenced to 3-(trimethylsilyl)-propane sulfonic acid Na-salt (TSPSA) defined as 0.00 ppm. Signals from two rotameric forms in equilibrium (E/Z isomerism) were obtained from the anion form of the compounds, and only the major form was used.

A training set of five quinoline derivatives with structural diversification performed at position 5 of the quinoline ring system was used for the SAR. PCA was used to uncover any relationship between the binding activities of the quinoline derivatives at the S100 proteins and the inhibitory effect of these derivatives in the aEAE model. PLS was then used to model the relationship between the physiochemical descriptors used to characterize the compounds and their biological responses. The PCA included the binding affinities towards murine and human S100A9 homodimers, murine S100A8/A9 heterodimer, and 50% effective dose (ED_50_) values from an aEAE mice model. The PLS analysis included binding affinity from the murine S100A9 homodimer and a total of 13 physicochemical variables used to describe the same set of five compounds. All variables were mean centred and scaled to unit variance.

## Supporting Information

Figure S1 S100A9 Interacts Specifically with TLR4 in a LPS Independent Manner (A) 100 nM hS100A9 was injected (for 3 min at 30 μl/min) over immobilized hTLR4/MD2 with soluble hTLR4/MD2 and LPS, 200 ng/ml, as competitors. HBS-P containing 10 μM Zn^2+^ and 1 mM Ca^2+^ was used as sample and running buffer. Responses at late association phase were calculated and plotted in a histogram. Inhibition of S100A9 binding was obtained with 200 and 400 nM sTLR4/MD2 (55 and 78% inhibition, respectively). LPS at 200 ng/ml had no effect when injected with hS100A9 alone or with a combination of hS100A9 and hTLR4/MD2. In order to test whether S100A9 inhibition was TLR4 specific, rhMD-2 at 200 and 400 nM was injected together with hS100A9. No displacement of S100A9 binding to the immobilized hTLR4/MD2 was obtained with MD2 (unpublished data).
(B) In a parallel experiment, 100 nM mouse S100A9 was injected over a surface with amine coupled mTLR4/MD2 fusion protein (mLPS-Trap) with LPS and mLPS-Trap as competitors. Mouse TLR4/MD2 at 100, 200, and 400 nM displaced mS100A9 binding in a dose-dependent manner (yielding 22%, 51%, and 70% inhibition, respectively), whereas LPS had no effect on mS100A9 binding.
(119 KB PDF)

Figure S2 Predicted versus Observed Binding of Compounds Listed in Table S1
(229 KB PDF)

Figure S3 Specificity Profile of the 43/8 Monoclonal Antibody (A) Dot-blot analysis of 43/8 reactivity to the indicated proteins.
(B) Denaturing gel electrophoresis of anti-S100A9 antibody 43/8 before and after Fab fragmentation. Lanes 1 and 6: 0.5 μg of mAb 43/8 with and without DTT; lanes 2 and 3: 1.0 and 0.5 μg of Fab 43/8 with DTT; and lanes 4 and 5: 0.5 and 1.0 μg Fab 43/8 without DTT. Molecular weight standards (Mw Std) from top to bottom: 250, 150, 100, 75, 50, 37, 25, 20, 15, and 10 k. Applied sample volume : 30 μl/well. Arrows indicate estimated molecular weights of mAb and Fab ± reduction.
(2.67 MB PDF)

Figure S4 Binding of hS100A9 to hRAGE/Fc and hTLR4/MD2 Is Not Due to hFc or hMD2 (A) 100 nM RAGE/Fc or Fc was injected over hS100A9 immobilized via the SH-group of the only cysteine in position 3 (left panel).
(B) Left: 100 nM TLR4/MD2 or MD2 injected over amino-coupled hS100A9. Fit of sensorgrams to a 1:1 model with mass transfer gave K_D_ of 3.4 and 9.2 nM and maximum responses at 352 and 214 relative units (RU) for RAGE and TLR4, respectively. Fab 43/8 was injected at 50–200 nM to verify activity of immobilized hS100A9 (right). Although the coupling density was high (∼5,000 RU), the 43/8 Fab demonstrated low but dose-dependent binding. This suggests that the RAGE and TLR4 binding activity of S100A9 is impaired by presentation on a solid phase.
(482 KB PDF)

Figure S5 Titration Curve Showing the Influence of Ca^2+^ on hS100A9 Binding to Immobilized ABR-224649 hS100A9 was injected at 100 nM at Ca^2+^ concentrations varying from 0 to 500 μM in the absence (open diamonds) or presence (filled squares) of 10 μM Zn^2+^. Responses were calculated at late association phase and plotted versus the concentration of Ca^2+^. Very similar results were obtained when hS100A9 was injected over immobilized RAGE or TLR4 and if Zn^2+^ was titrated in the absence or presence of Ca^2+^ (unpublished data).
(234 KB PDF)

Figure S6 Inhibition of hS100A9 Binding to Immobilized hRAGE, Q Compound, and hTLR4 by ABR-215757 and a Negative Control (Substance Lacking the Keto-Enol Group of Q Compounds) *B*_comp_/*B*_0_ in percentages was plotted versus concentration of competitor.
(267 KB PDF)

Figure S7 hS100A9 Binding to TLR4/MD2 Is Proportional to the Coupling Density of TLR4 on the Chip S100A9 was injected at 100 nM over TLR4/MD2 immobilized by amine coupling at three densities. Sensorgrams from bottom to top represent an immobilization level of 770, 1,400, and 2,250 RU, respectively. Similar results were obtained when RAGE was coupled at varying densities (unpublished data).
(255 KB PDF)

Figure S8 EAE Was Induced in S100A9^−/−^(KO) and Normal Littermate Controls (Third Back-Cross Generation to C57BL/6) Using MOG Peptide The animals were randomized to treatment with ABR-215757 (KO: *n* = 5, 3 females/2 males), (wild type [wt]: *n* = 6, 3 females/3 males) or normal drinking water (KO: *n* = 5, 3 females/2 males), (wt: *n* = 6, 3 females/3 males). An experiment performed using animals from the second back-cross generation to C57BL/6 provided similar results.
(24 KB PDF)

Table S1 Summary of Data Used for SAR Analysis To the left, the compound name is defined and the substitution in the R5 position is specified, as well as the data obtained for the various molecular interactions and in vivo aEAE data.
(43 KB PDF)

## Abbreviations

aEAE: acute experimental autoimmune encephalomyelitis
LPS: lipopolysaccharide
PCA: principal component analysis
PLS: partial least squares projections to latent structures
RU: relative unit
SAR: structure-activity relationship
SPR: surface plasmon resonance
