# Supplementary material for: Identification of Human S100A9 as a Novel Target for Treatment of Autoimmune Disease via Binding to Quinoline-3-Carboxamides
Source: PLoS Biol. 2009 Apr 28;7(4):e1000097. doi: 10.1371/journal.pbio.1000097 (PMC2671563; doi:10.1371/journal.pbio.1000097)
Supplement: Supplementary file 1 [file pbio.1000097.sg001.pdf]

A

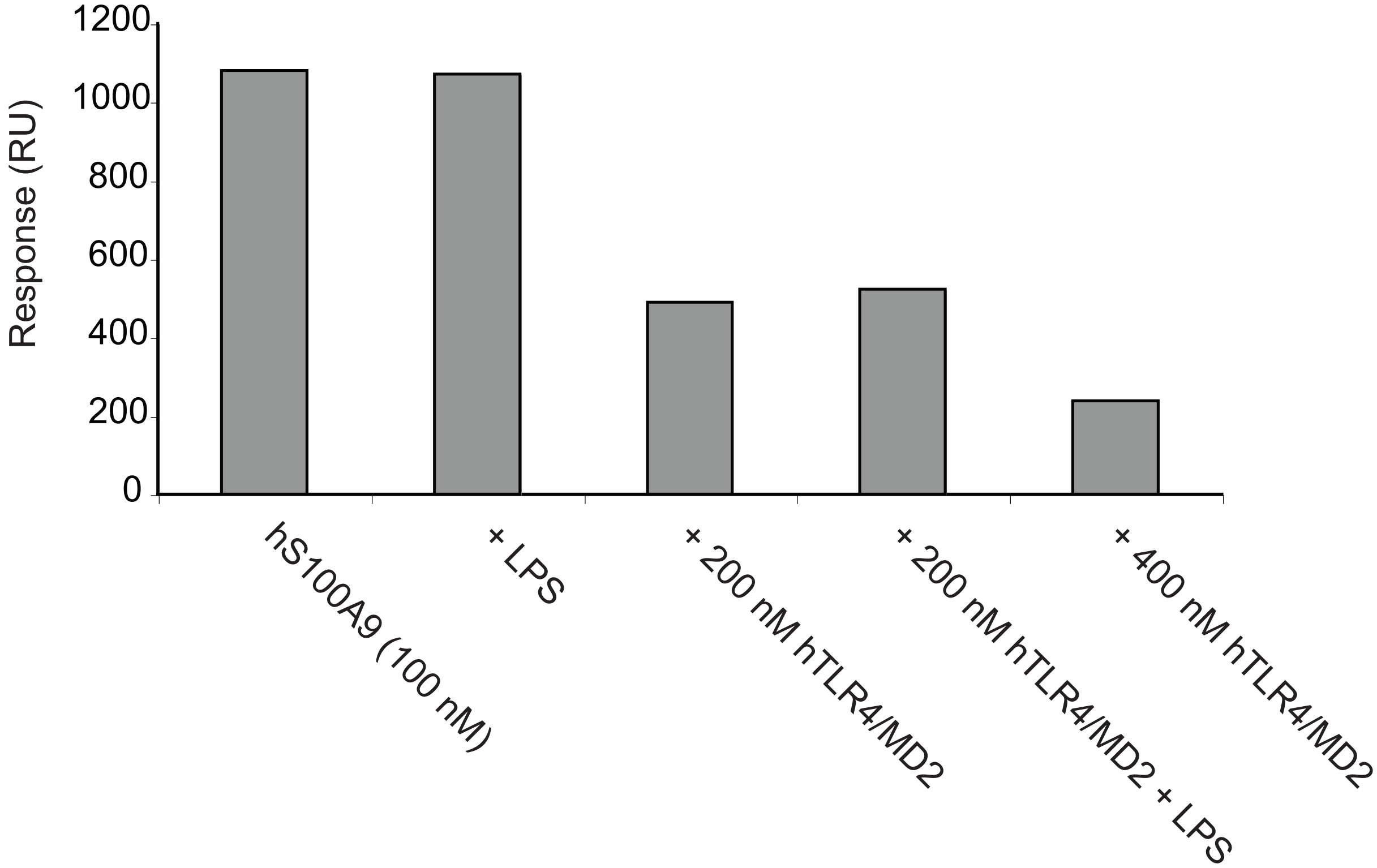
1200
1000
800
600
400
200
0
Response (RU)
hS100A9 (100 nM)
+ LPS
+ 200 nM hTLR4/MD2
+ 200 nM hTLR4/MD2 + LPS
+ 400 nM hTLR4/MD2

B

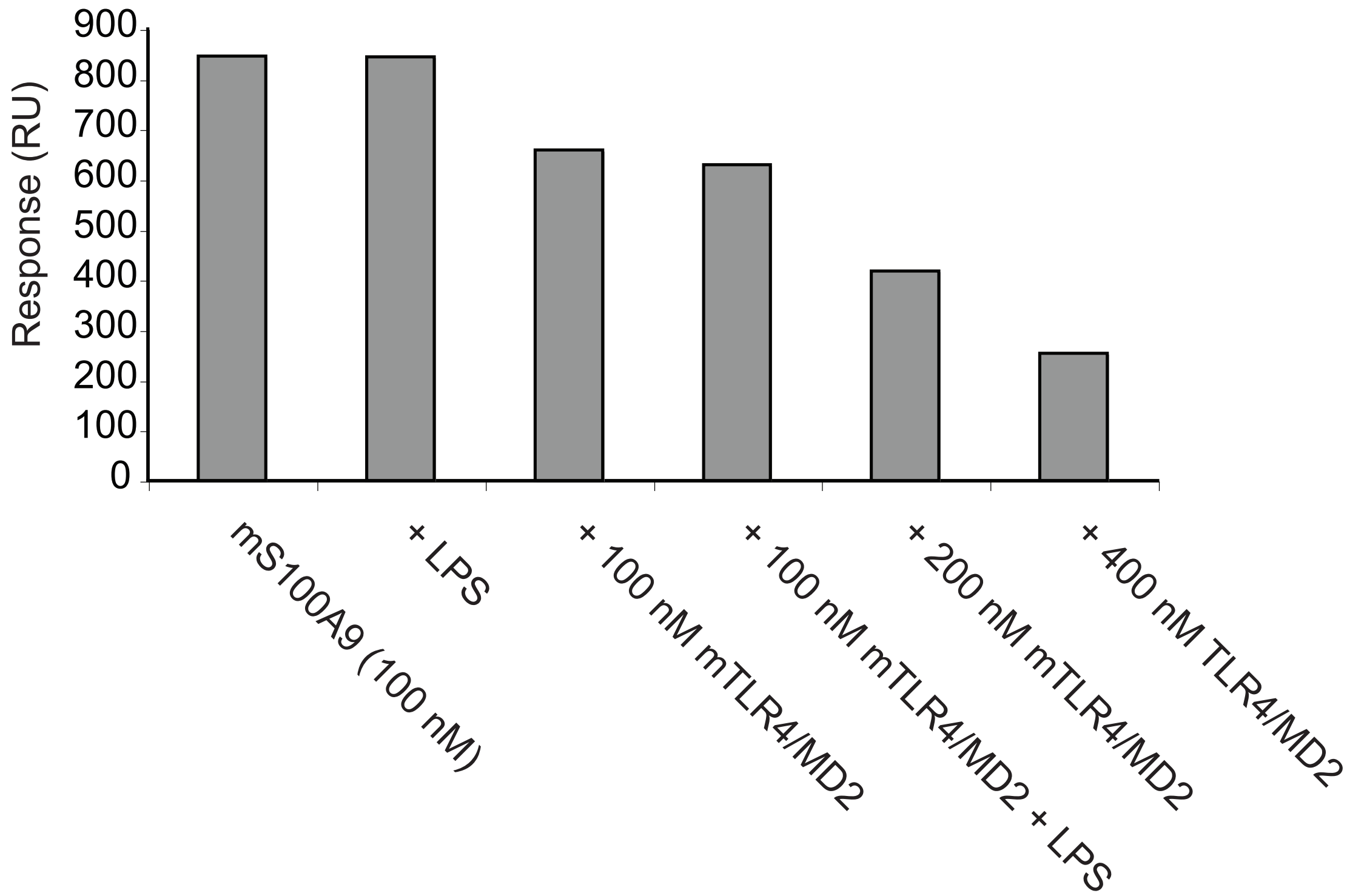
900
800
700
600
500
400
300
200
100
0
Response (RU)
mS100A9 (100 nM)
+ LPS
+ 100 nM mTLR4/MD2
+ 100 nM mTLR4/MD2 + LPS
+ 200 nM mTLR4/MD2
+ 400 nM TLR4/MD2
