# Supplementary material for: Identification of Human S100A9 as a Novel Target for Treatment of Autoimmune Disease via Binding to Quinoline-3-Carboxamides
Source: PLoS Biol. 2009 Apr 28;7(4):e1000097. doi: 10.1371/journal.pbio.1000097 (PMC2671563; doi:10.1371/journal.pbio.1000097)

Supplementary Figure 2

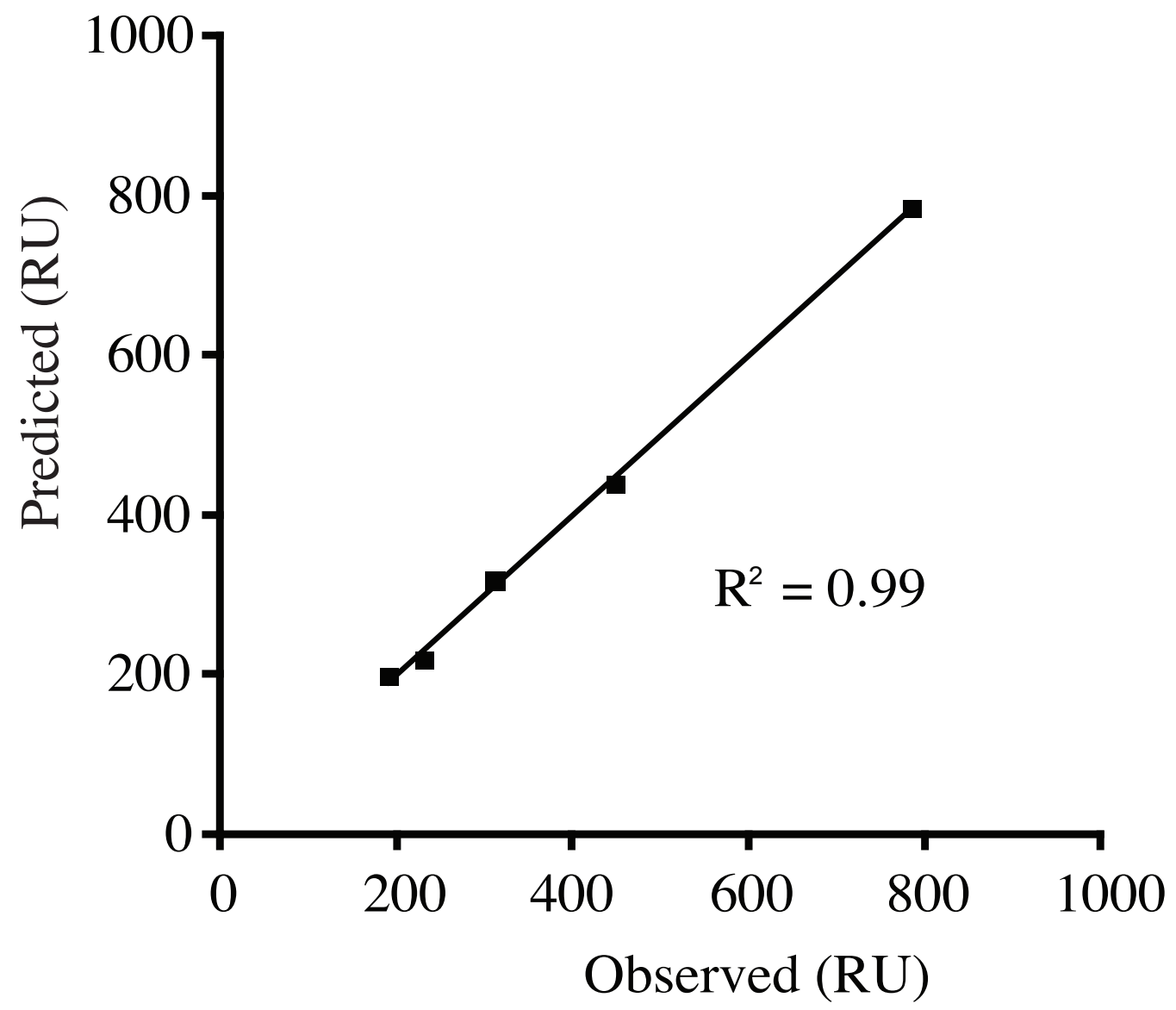

Supplement: Supplementary file 2 [file pbio.1000097.sg002.pdf]
