# Supplementary material for: Identification of Human S100A9 as a Novel Target for Treatment of Autoimmune Disease via Binding to Quinoline-3-Carboxamides
Source: PLoS Biol. 2009 Apr 28;7(4):e1000097. doi: 10.1371/journal.pbio.1000097 (PMC2671563; doi:10.1371/journal.pbio.1000097)
Supplement: Supplementary file 3 [file pbio.1000097.sg003.pdf]

A

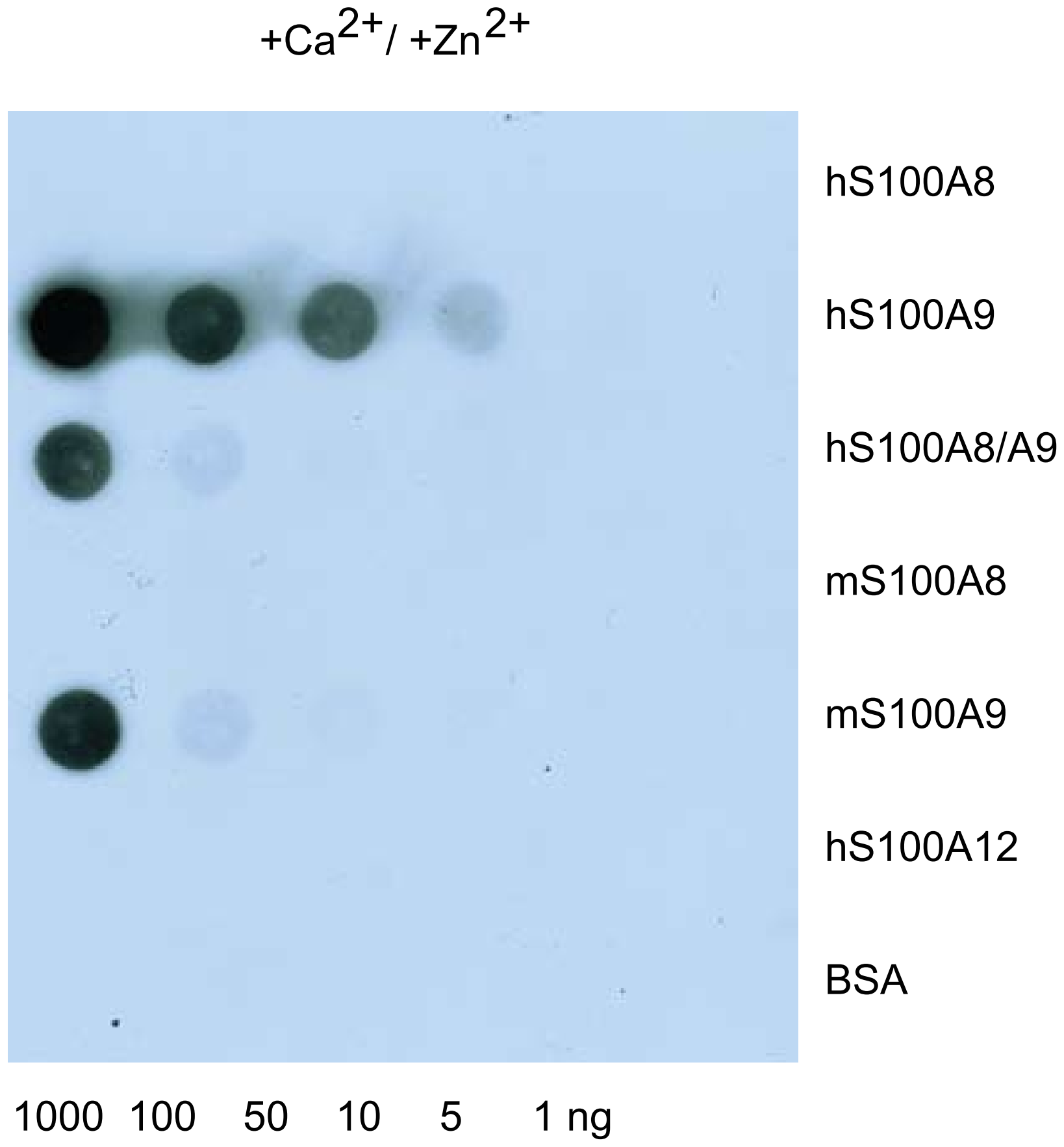
+Ca2+/ +Zn2+
hS100A8
hS100A9
hS100A8/A9
mS100A8
mS100A9
hS100A12
BSA
1000 100 50 10 5 1 ng

B

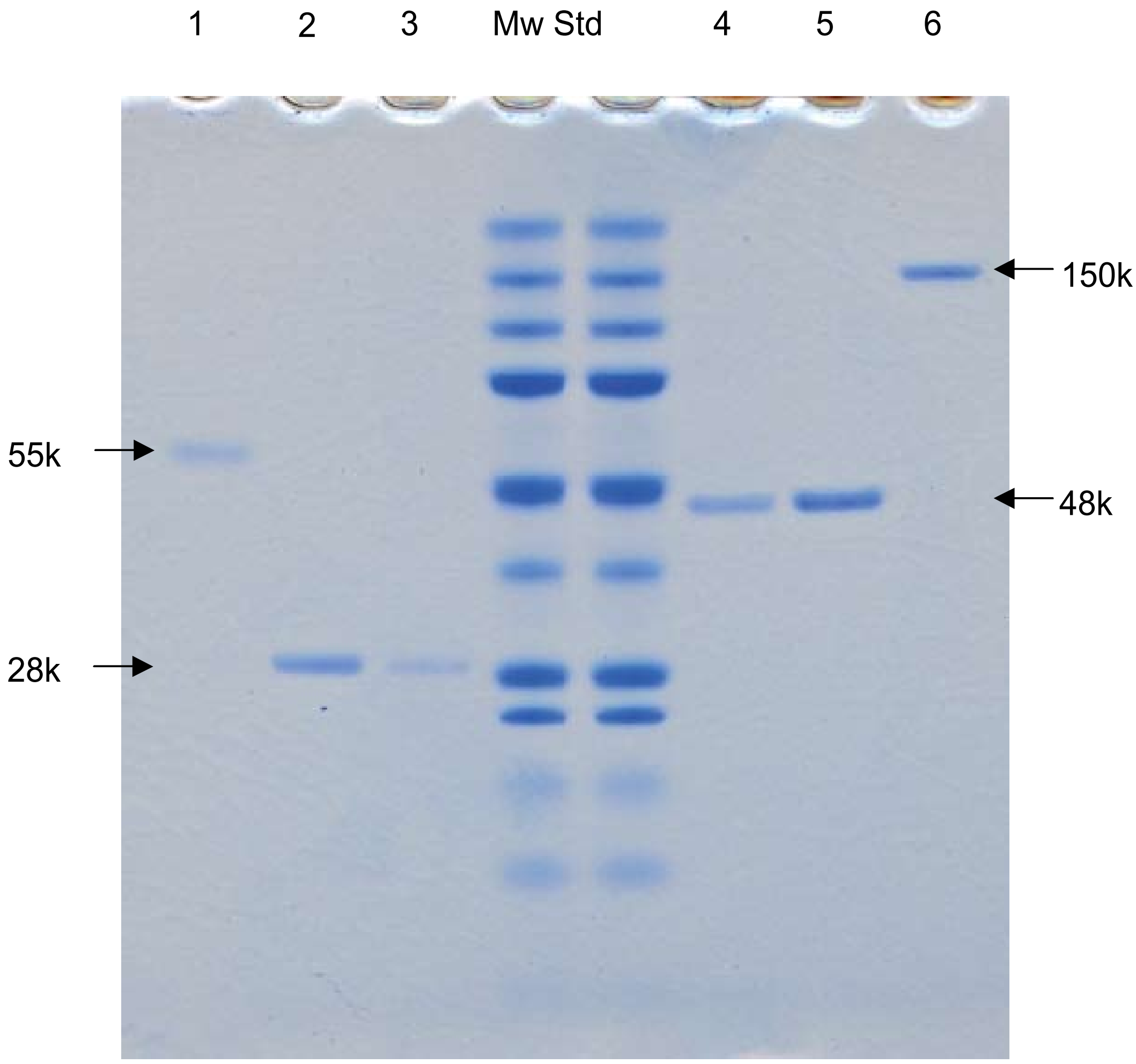
1
2
3
Mw Std
4
5
6
55k
28k
150k
48k
