# Supplementary material for: Identification of Human S100A9 as a Novel Target for Treatment of Autoimmune Disease via Binding to Quinoline-3-Carboxamides
Source: PLoS Biol. 2009 Apr 28;7(4):e1000097. doi: 10.1371/journal.pbio.1000097 (PMC2671563; doi:10.1371/journal.pbio.1000097)
Supplement: Supplementary file 4 [file pbio.1000097.sg004.pdf]

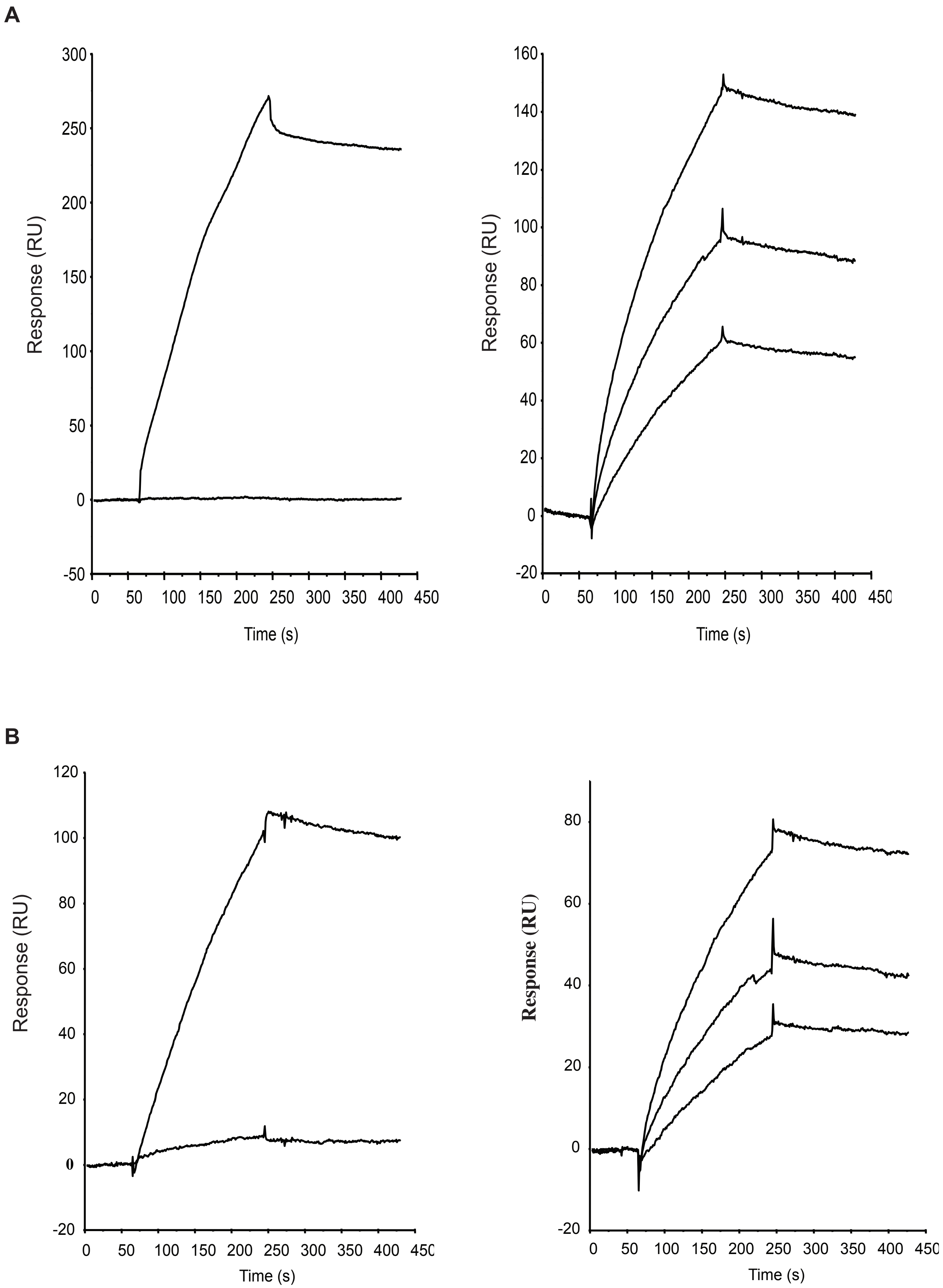
A
Response (RU)
Time (s)
300
250
200
150
100
50
0
-50
160
140
120
100
80
60
40
20
0
-20
0 50 100 150 200 250 300 350 400 450
B
Response (RU)
Time (s)
120
100
80
60
40
20
0
-20
0 50 100 150 200 250 300 350 400 450
