# Supplementary material for: Identification of Human S100A9 as a Novel Target for Treatment of Autoimmune Disease via Binding to Quinoline-3-Carboxamides
Source: PLoS Biol. 2009 Apr 28;7(4):e1000097. doi: 10.1371/journal.pbio.1000097 (PMC2671563; doi:10.1371/journal.pbio.1000097)
Supplement: Supplementary file 5 [file pbio.1000097.sg005.pdf]

ABR-224649

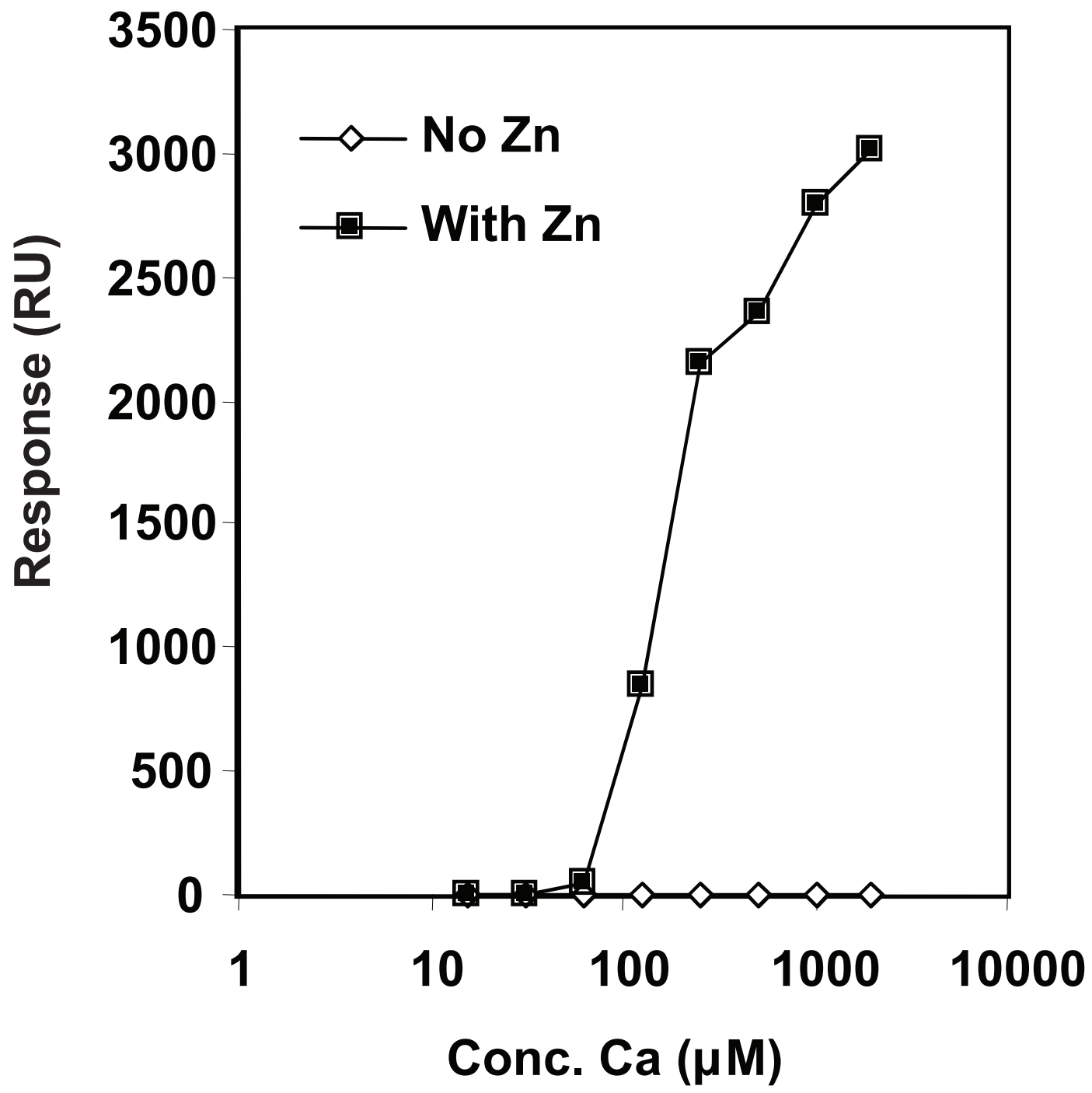

Response (RU)
3500
3000
2500
2000
1500
1000
500
0
No Zn
With Zn
1
10
100
1000
10000
Conc. Ca (μM)
