# Supplementary material for: Identification of Human S100A9 as a Novel Target for Treatment of Autoimmune Disease via Binding to Quinoline-3-Carboxamides
Source: PLoS Biol. 2009 Apr 28;7(4):e1000097. doi: 10.1371/journal.pbio.1000097 (PMC2671563; doi:10.1371/journal.pbio.1000097)
Supplement: Supplementary file 6 [file pbio.1000097.sg006.pdf]

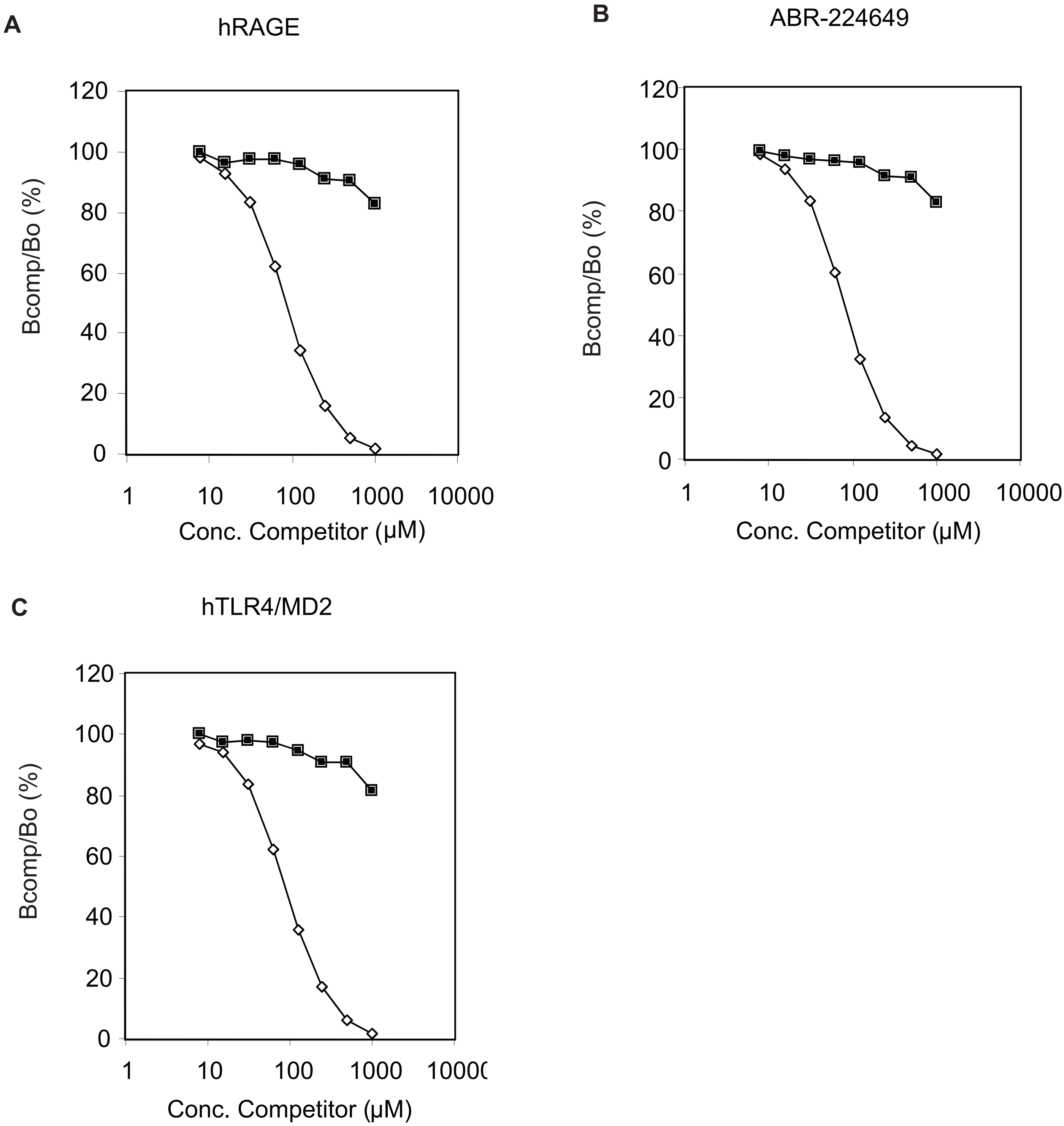
A
hRAGE
120
100
80
60
40
20
0
Bcomp/Bo (%)
1
10
100
1000
10000
Conc. Competitor (μM)
B
ABR-224649
120
100
80
60
40
20
0
Bcomp/Bo (%)
1
10
100
1000
10000
Conc. Competitor (μM)
C
hTLR4/MD2
120
100
80
60
40
20
0
Bcomp/Bo (%)
1
10
100
1000
1000(
Conc. Competitor (μM)
