# Supplementary material for: Identification of Human S100A9 as a Novel Target for Treatment of Autoimmune Disease via Binding to Quinoline-3-Carboxamides
Source: PLoS Biol. 2009 Apr 28;7(4):e1000097. doi: 10.1371/journal.pbio.1000097 (PMC2671563; doi:10.1371/journal.pbio.1000097)
Supplement: Supplementary file 8 [file pbio.1000097.sg008.pdf]

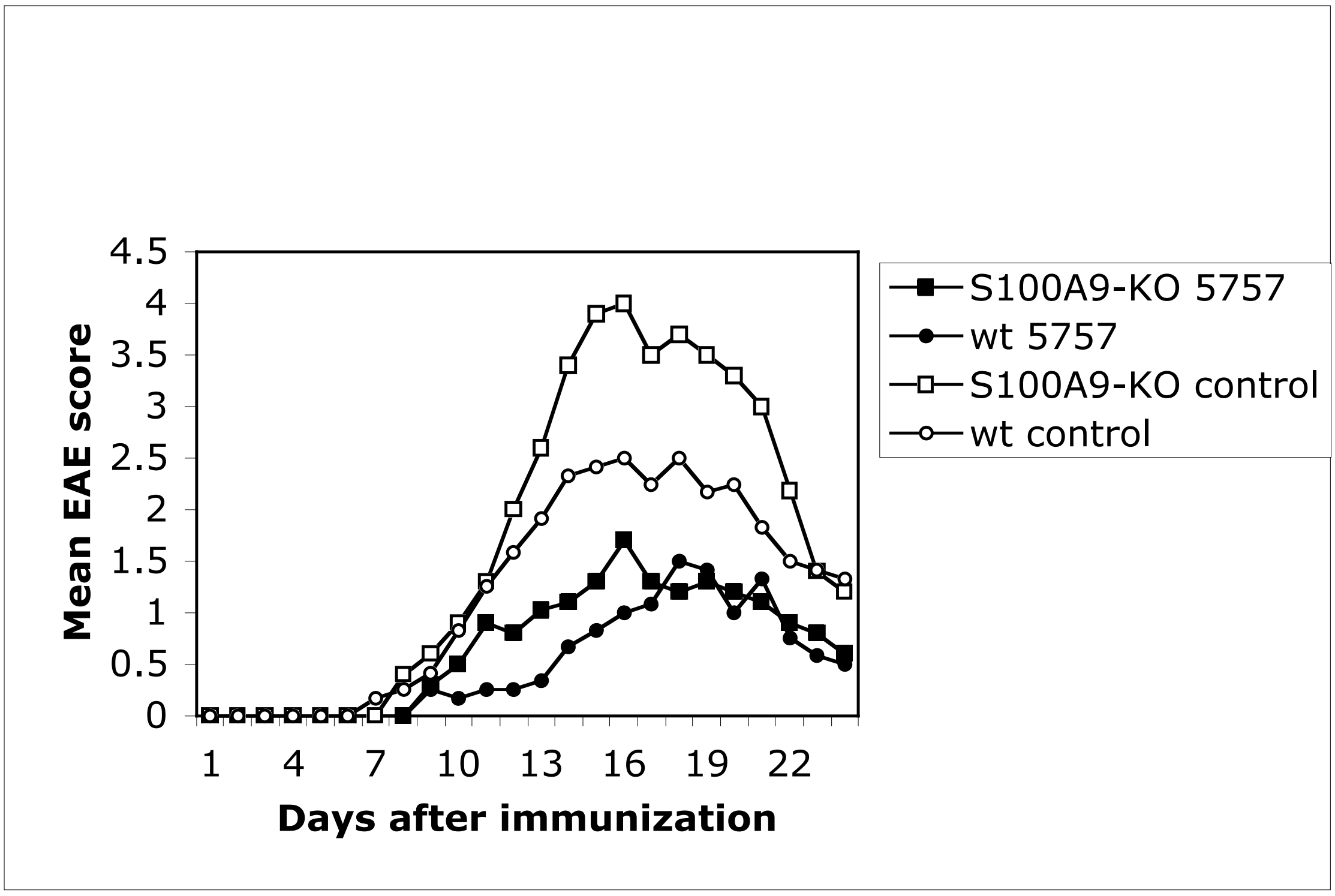

Mean EAE score
4.5
4
3.5
3
2.5
2
1.5
1
0.5
0
1
4
7
10
13
16
19
22
Days after immunization
S100A9-KO 5757
wt 5757
S100A9-KO control
wt control
