# Supplementary material for: Identification of Human S100A9 as a Novel Target for Treatment of Autoimmune Disease via Binding to Quinoline-3-Carboxamides
Source: PLoS Biol. 2009 Apr 28;7(4):e1000097. doi: 10.1371/journal.pbio.1000097 (PMC2671563; doi:10.1371/journal.pbio.1000097)
Supplement: Supplementary file 9 [file pbio.1000097.st001.pdf]

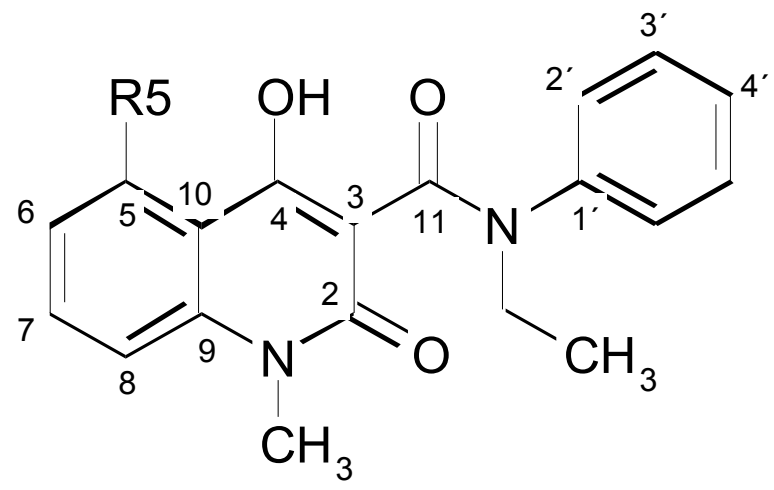

3-Quinoline carboxamides and their S100A9 binding ability and effects on aEAE development in SLJ/N mice.

ABR-21#	R5	S100A9 binding IC_{50} (μM)					aEAE Inhibition
		hS100A9		mS100A9			ED_{50} (mg/kg)
		RAGE	TLR4	Inhibition	RAGE	TLR4	
2662	H	436	415	1936	1026	1972	1.87
5111	CH_3	168	217	1337	783	1236	0.55
5628	SCH_3	135	154	906	447	780	0.20
5629	CF_3	68,5	79	916	230	741	0.13
5727	Br	78,7	87,9	522	191	440	0.16
5757	CH_2CH_3	60,5	59,7	940	311	698	0.04

3-Quinoline carboxamides and their S100A8/A9 binding ability.

ABR-21#	R5	S100A8-9 binding IC_{50} (μM)	
		mS100A8/A9	
		RAGE	TLR4
2662	H	720	576
5111	CH_3	310	293
5628	SCH_3	174	257
5629	CF_3	351	301
5727	Br	164	200
5757	CH_2CH_3	334	334
